# (*E*)-*N*-(Anthracen-9-yl­methyl­idene)-4-nitro­aniline

**DOI:** 10.1107/S1600536811035859

**Published:** 2011-09-14

**Authors:** K. Geetha, D. K. Andrew Prasanna Kumar, D. Lakshmanan, R. Savitha, S. Murugavel

**Affiliations:** a Department of Chemistry, SRM University, Vadapalani Campus, Chennai 600 026, India; b Department of Physics, Voorhees College, Vellore 632 001, India; c Department of Physics, C. Abdul Hakeem College of Engineering & Technology, Melvisharam, Vellore 632 509, India; d Department of Physics, Thanthai Periyar Government Institute of Technology, Vellore 632 002, India.

## Abstract

In the title molecule, C_21_H_14_N_2_O_2_, the anthracenyl system is approximately planar [maximum deviation = 0.056 (4) Å] and is oriented at a dihedral angle of 73.6 (1)° with respect to the benzene ring. An intra­molecular C—H⋯N hydrogen bond generates an *S*(6) ring motif. The crystal packing is stabilized by C—H⋯π and π–π inter­actions [centroid–centroid distances of 3.688 (2), 3.656 (1) and 3.716 (2) Å].

## Related literature

For applications of anthracene derivatives, see: de Silva *et al.* (1997 ▶); Klarner *et al.* (1998 ▶); Han *et al.* (2009 ▶). For hydrogen-bond motifs, see: Bernstein *et al.* (1995 ▶). For related structures, see: Arumugam *et al.* (2011 ▶); Villalpando *et al.* (2010 ▶).
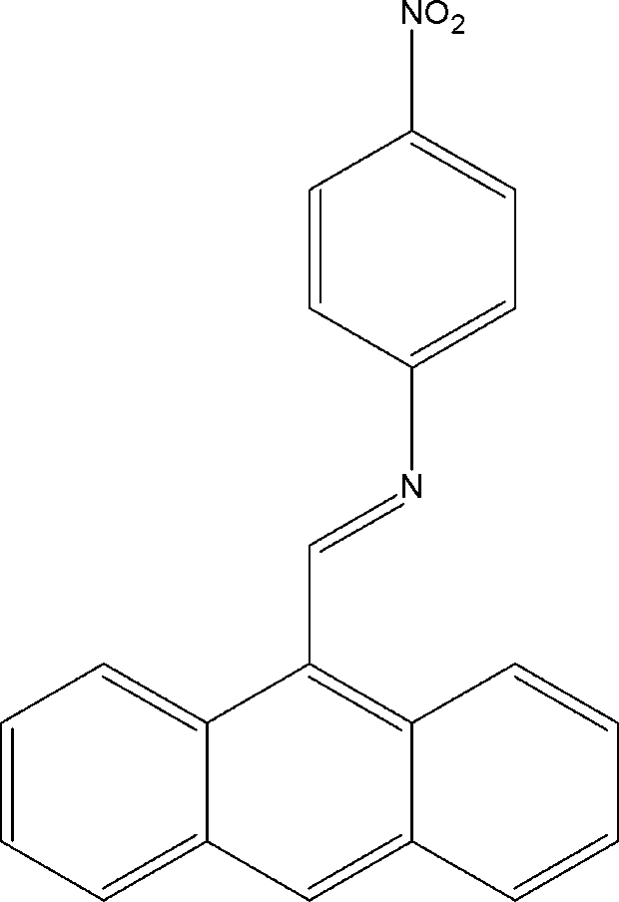


## Experimental

### 

#### Crystal data

C_21_H_14_N_2_O_2_
*M* *_r_* = 326.34
Triclinic, 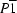

*a* = 8.3634 (4) Å
*b* = 8.9045 (4) Å
*c* = 11.5119 (6) Å
α = 75.235 (2)°
β = 84.544 (3)°
γ = 75.054 (2)°
*V* = 800.56 (7) Å^3^
*Z* = 2
Mo *K*α radiation
μ = 0.09 mm^−1^
*T* = 293 K
0.30 × 0.20 × 0.10 mm

#### Data collection

Bruker APEXII diffractometer
Absorption correction: multi-scan (*SADABS*; Bruker 2004 ▶) *T*_min_ = 0.924, *T*_max_ = 0.991
15391 measured reflections
2983 independent reflections
1870 reflections with *I* > 2σ(*I*)
*R*_int_ = 0.151

#### Refinement

*R*[*F* ^2^ > 2σ(*F* ^2^)] = 0.070
*wR*(*F* ^2^) = 0.306
*S* = 1.12
2983 reflections
226 parameters
H-atom parameters constrained
Δρ_max_ = 0.45 e Å^−3^
Δρ_min_ = −0.43 e Å^−3^

### 

Data collection: *APEX2* (Bruker, 2004 ▶); cell refinement: *APEX2* and *SAINT* (Bruker, 2004 ▶); data reduction: *SAINT* and *XPREP* (Bruker, 2004 ▶); program(s) used to solve structure: *SHELXS97* (Sheldrick, 2008 ▶); program(s) used to refine structure: *SHELXL97* (Sheldrick, 2008 ▶); molecular graphics: *ORTEP-3* (Farrugia (1997 ▶); software used to prepare material for publication: *SHELXL97* and *PLATON* (Spek, 2009 ▶).

## Supplementary Material

Crystal structure: contains datablock(s) global, I. DOI: 10.1107/S1600536811035859/im2315sup1.cif

Structure factors: contains datablock(s) I. DOI: 10.1107/S1600536811035859/im2315Isup2.hkl

Additional supplementary materials:  crystallographic information; 3D view; checkCIF report

## Figures and Tables

**Table 1:** Hydrogen-bond geometry (Å, °) *Cg*1 is the centroid of the C1–C6 benzene ring.

*D*—H⋯*A*	*D*—H	H⋯*A*	*D*⋯*A*	*D*—H⋯*A*
C12—H12⋯N1	0.93	2.37	2.980 (4)	123
C20—H20⋯*Cg*1^i^	0.93	2.86	3.717 (3)	154

Symmetry code: (i) 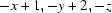.

**Fig. 1.**
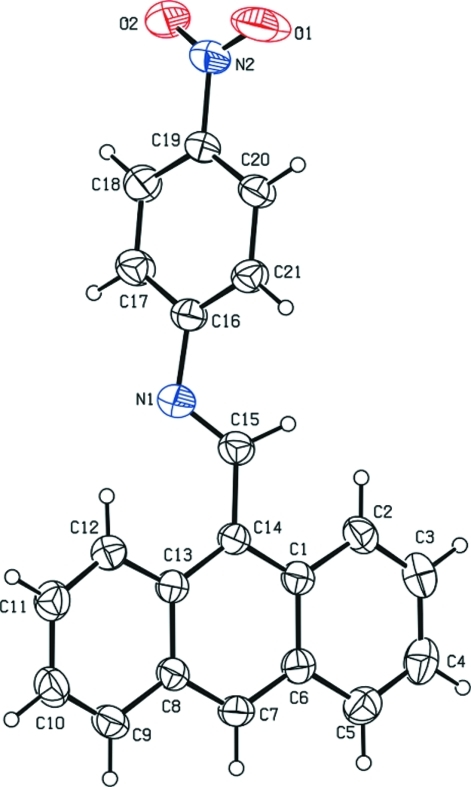
Molecular structure of the title compound with the atom numbering scheme. Displacement ellipsoids are drawn at the 30% probability level. H atoms are presented as a small cycles of arbitrary radius.

**Fig. 2.**
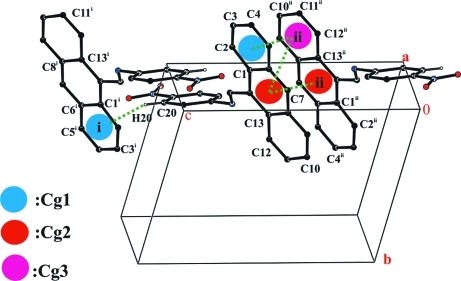
Intramolecular C-H···O, C-H···π and π-π interactions (dotted lines) in the title compound. Cg1, Cg2 and Cg3 are the centroids of the C1-C6 benzene ring, C1/C6/C7/C8/C13/C14 benzene ring and C8-C13 benzene ring , respectively. [Symmetry code: (i) 1-x, -y, 2-z; (ii) 1-x, -y, 1-z.]

## supplementary crystallographic information

### Comment

Anthracene is an attractive material in its photochemical and electrochemical properties and is used as a potential medium for photoconductive (de Silva *et al.*, 1997) and electroluminescence (Klarner *et al.*, 1998) devices. Furthermore, anthracene derivatives exhibited anticancer activity has also been reported recently (Han *et al.*, 2009). Against this background and in order to obtain detailed information on molecular conformations in the solid state, X-ray studies of the title compound have been carried out.

Fig. 1. shows a displacement ellipsoid plot of (I), with the atom numbering scheme. The anthracene moiety (C1-C14) is essentially planar [maximum deviation = -0.056 (4) Å for the C11 atom] and shows a dihedral angle of 73.6 (1)° with respect to the (C16-C21) benzene ring. The nitro group is slightly twisted away from the plane of the attached benzene ring [C20-C19-N2-O1 = -4.9 (5) ° and C18-C19-N2-O2 = -6.7 (5) °]. The geometric parameters of the title molecule agrees well with those reported for similar structures (Arumugam *et al.*, 2011, Villalpando *et al.*, 2010).

In addition to van der Waals interactions, the crystal packing is stabilized by C-H···N and C-H···π hydrogen bonds as well as by π-π interactions. The intramolecular C12-H12···N1 hydrogen bond generates an S(6) ring motif (Bernstein *et al.*, 1995). The crystal packing (Fig. 2) is stabilized by C-H···π interactions between H20 and the neighbouring C1-C6 benzene ring, with a C20-H20···Cg1^i^ separation of 2.86 Å (Fig. 2, Table 1; Cg1 is the centroid of the C1-C6 benzene ring, symmetry code as in Fig. 2). The molecular packing (Fig. 2) is further stabilized by π-π interactions with Cg1···Cg3^ii^, Cg2···Cg2^ii^ and Cg2···Cg3^ii^ separations of 3.688 (2) Å, 3.656 (1) Å and 3.716 (2) Å, respectively (Fig. 2; Cg1, Cg2 and Cg3 are the centroids of the C1-C6 benzene ring,C1/C6/C7/C8/C13/C14 benzene ring and C8-C13 benzene ring , respectively, symmetry code as in Fig. 2).

### Experimental

Equimolar amounts of p-nitroaniline and 9-anthracenecarboxaldehyde were suspended in ethanol at a concentration of 0.1 M and the reaction mixture was refluxed overnight under vigorous stirring. Afterwards the mixture was cooled down and filtered. Recrystallization of the crude product from hexane : CHCl_3_ (1 : 1) yielded orange crystals of title compound (Yield 74 %).

### Refinement

All H atoms were positioned geometrically, with C-H = 0.93 - 0.98 Å and constrained to ride on their parent atom with *U*_iso_(H)=1.2*U*_eq_(C).

### Crystal data

**Table:**

C_21_H_14_N_2_O_2_	*Z* = 2
*M**_r_* = 326.34	*F*(000) = 340
Triclinic, *P*1	*D*_x_ = 1.354 Mg m^−^^3^
Hall symbol: -P1	Mo *K*α radiation, λ = 0.71073 Å
*a* = 8.3634 (4) Å	Cell parameters from 5007 reflections
*b* = 8.9045 (4) Å	θ = 2.5–25.3°
*c* = 11.5119 (6) Å	µ = 0.09 mm^−^^1^
α = 75.235 (2)°	*T* = 293 K
β = 84.544 (3)°	Flat, orange
γ = 75.054 (2)°	0.30 × 0.20 × 0.10 mm
*V* = 800.56 (7) Å^3^	

### Data collection

**Table:**

Bruker APEXII diffractometer	2983 independent reflections
Radiation source: fine-focus sealed tube	1870 reflections with *I* > 2σ(*I*)
graphite	*R*_int_ = 0.151
ω and φ scan	θ_max_ = 25.6°, θ_min_ = 2.5°
Absorption correction: multi-scan (*SADABS*; Bruker 2004)	*h* = −10→10
*T*_min_ = 0.924, *T*_max_ = 0.991	*k* = −10→10
15391 measured reflections	*l* = −13→13

### Refinement

**Table:**

Refinement on *F*^2^	Primary atom site location: structure-invariant direct methods
Least-squares matrix: full	Secondary atom site location: difference Fourier map
*R*[*F*^2^ > 2σ(*F*^2^)] = 0.070	Hydrogen site location: inferred from neighbouring sites
*wR*(*F*^2^) = 0.306	H-atom parameters constrained
*S* = 1.12	*w* = 1/[σ^2^(*F*_o_^2^) + (0.2*P*)^2^] where *P* = (*F*_o_^2^ + 2*F*_c_^2^)/3
2983 reflections	(Δ/σ)_max_ < 0.001
226 parameters	Δρ_max_ = 0.45 e Å^−^^3^
0 restraints	Δρ_min_ = −0.43 e Å^−^^3^

### Special details

**Table:**

Geometry. All esds (except the esd in the dihedral angle between two l.s. planes) are estimated using the full covariance matrix. The cell esds are taken into account individually in the estimation of esds in distances, angles and torsion angles; correlations between esds in cell parameters are only used when they are defined by crystal symmetry. An approximate (isotropic) treatment of cell esds is used for estimating esds involving l.s. planes.
Refinement. Refinement of F^2^ against ALL reflections. The weighted R-factor wR and goodness of fit S are based on F^2^, conventional R-factors R are based on F, with F set to zero for negative F^2^. The threshold expression of F^2^ > 2sigma(F^2^) is used only for calculating R-factors(gt) etc. and is not relevant to the choice of reflections for refinement. R-factors based on F^2^ are statistically about twice as large as those based on F, and R- factors based on ALL data will be even larger.

### Fractional atomic coordinates and isotropic or equivalent isotropic displacement parameters (Å^2^)

**Table:**

	*x*	*y*	*z*	*U*_iso_*/*U*_eq_	
C1	0.4268 (3)	−0.1323 (3)	0.7152 (2)	0.0507 (7)	
C2	0.5139 (4)	−0.2566 (4)	0.8071 (2)	0.0622 (8)	
H2	0.5912	−0.2354	0.8498	0.075*	
C3	0.4872 (4)	−0.4052 (4)	0.8341 (3)	0.0725 (9)	
H3	0.5463	−0.4840	0.8950	0.087*	
C4	0.3730 (4)	−0.4430 (4)	0.7726 (3)	0.0746 (10)	
H4	0.3553	−0.5455	0.7936	0.090*	
C5	0.2885 (4)	−0.3314 (4)	0.6829 (3)	0.0673 (9)	
H5	0.2134	−0.3579	0.6417	0.081*	
C6	0.3125 (3)	−0.1725 (3)	0.6501 (2)	0.0532 (7)	
C7	0.2314 (3)	−0.0591 (3)	0.5551 (2)	0.0549 (8)	
H7	0.1599	−0.0875	0.5121	0.066*	
C8	0.2524 (3)	0.0973 (3)	0.5211 (2)	0.0498 (7)	
C9	0.1657 (3)	0.2116 (4)	0.4241 (2)	0.0621 (8)	
H9	0.0979	0.1808	0.3798	0.075*	
C10	0.1794 (4)	0.3636 (4)	0.3950 (3)	0.0703 (9)	
H10	0.1211	0.4375	0.3312	0.084*	
C11	0.2830 (4)	0.4104 (4)	0.4619 (3)	0.0675 (9)	
H11	0.2904	0.5164	0.4427	0.081*	
C12	0.3710 (3)	0.3050 (3)	0.5525 (2)	0.0588 (8)	
H12	0.4395	0.3393	0.5939	0.071*	
C13	0.3618 (3)	0.1421 (3)	0.5868 (2)	0.0487 (7)	
C14	0.4501 (3)	0.0265 (3)	0.6825 (2)	0.0479 (7)	
C15	0.5698 (3)	0.0602 (4)	0.7521 (3)	0.0584 (8)	
H15	0.5835	0.0005	0.8311	0.070*	
C16	0.7666 (3)	0.1767 (3)	0.7942 (2)	0.0553 (8)	
C17	0.9203 (4)	0.1906 (4)	0.7470 (3)	0.0687 (9)	
H17	0.9467	0.1877	0.6672	0.082*	
C18	1.0355 (4)	0.2085 (4)	0.8158 (3)	0.0695 (9)	
H18	1.1407	0.2147	0.7841	0.083*	
C19	0.9936 (3)	0.2169 (3)	0.9316 (2)	0.0561 (8)	
C20	0.8419 (3)	0.2054 (4)	0.9826 (2)	0.0597 (8)	
H20	0.8168	0.2105	1.0622	0.072*	
C21	0.7263 (3)	0.1859 (3)	0.9128 (2)	0.0589 (8)	
H21	0.6214	0.1791	0.9451	0.071*	
N1	0.6547 (3)	0.1602 (3)	0.7163 (2)	0.0647 (7)	
N2	1.1188 (4)	0.2349 (4)	1.0045 (3)	0.0823 (9)	
O1	1.0791 (4)	0.2499 (5)	1.1057 (3)	0.1296 (12)	
O2	1.2591 (3)	0.2269 (4)	0.9629 (2)	0.1103 (11)	

### Atomic displacement parameters (Å^2^)

**Table:**

	*U*^11^	*U*^22^	*U*^33^	*U*^12^	*U*^13^	*U*^23^
C1	0.0442 (15)	0.0651 (17)	0.0434 (14)	−0.0129 (12)	0.0062 (11)	−0.0174 (12)
C2	0.0615 (18)	0.076 (2)	0.0457 (15)	−0.0128 (14)	−0.0012 (13)	−0.0120 (13)
C3	0.087 (2)	0.070 (2)	0.0511 (17)	−0.0134 (17)	0.0044 (16)	−0.0059 (14)
C4	0.094 (3)	0.0552 (19)	0.073 (2)	−0.0245 (17)	0.0155 (18)	−0.0117 (15)
C5	0.0660 (19)	0.073 (2)	0.0698 (19)	−0.0249 (15)	0.0068 (15)	−0.0235 (16)
C6	0.0498 (16)	0.0619 (18)	0.0498 (15)	−0.0148 (13)	0.0074 (12)	−0.0187 (12)
C7	0.0490 (16)	0.0723 (19)	0.0503 (15)	−0.0204 (13)	−0.0009 (12)	−0.0218 (13)
C8	0.0412 (14)	0.0648 (17)	0.0423 (14)	−0.0115 (12)	0.0031 (11)	−0.0139 (12)
C9	0.0511 (17)	0.084 (2)	0.0502 (16)	−0.0143 (14)	−0.0074 (13)	−0.0139 (14)
C10	0.0645 (19)	0.076 (2)	0.0576 (18)	−0.0116 (15)	−0.0089 (15)	0.0030 (15)
C11	0.068 (2)	0.0588 (18)	0.0694 (19)	−0.0151 (15)	−0.0038 (15)	−0.0043 (14)
C12	0.0550 (17)	0.0650 (19)	0.0568 (17)	−0.0172 (13)	−0.0026 (13)	−0.0125 (13)
C13	0.0416 (14)	0.0623 (17)	0.0439 (14)	−0.0133 (12)	0.0062 (11)	−0.0176 (12)
C14	0.0405 (14)	0.0615 (17)	0.0428 (14)	−0.0133 (11)	0.0018 (11)	−0.0145 (12)
C15	0.0498 (16)	0.0717 (19)	0.0539 (16)	−0.0151 (14)	−0.0036 (12)	−0.0143 (13)
C16	0.0581 (17)	0.0556 (16)	0.0545 (16)	−0.0139 (12)	−0.0105 (13)	−0.0144 (12)
C17	0.0637 (19)	0.092 (2)	0.0553 (17)	−0.0213 (16)	0.0017 (14)	−0.0260 (15)
C18	0.0506 (17)	0.093 (2)	0.0633 (19)	−0.0197 (15)	0.0002 (14)	−0.0130 (15)
C19	0.0501 (16)	0.0639 (18)	0.0532 (16)	−0.0140 (13)	−0.0131 (13)	−0.0079 (12)
C20	0.0564 (18)	0.0771 (19)	0.0485 (15)	−0.0161 (14)	−0.0053 (13)	−0.0191 (13)
C21	0.0469 (15)	0.073 (2)	0.0600 (17)	−0.0187 (13)	0.0002 (13)	−0.0191 (14)
N1	0.0692 (16)	0.0745 (17)	0.0572 (14)	−0.0250 (13)	−0.0087 (12)	−0.0182 (12)
N2	0.068 (2)	0.113 (2)	0.0676 (18)	−0.0334 (16)	−0.0227 (15)	−0.0059 (16)
O1	0.100 (2)	0.225 (4)	0.093 (2)	−0.056 (2)	−0.0209 (17)	−0.069 (2)
O2	0.0675 (17)	0.169 (3)	0.097 (2)	−0.0584 (17)	−0.0212 (15)	0.0007 (17)

### Geometric parameters (Å, °)

**Table:**

C1—C2	1.418 (4)	C11—H11	0.9300
C1—C14	1.428 (4)	C12—C13	1.423 (4)
C1—C6	1.432 (4)	C12—H12	0.9300
C2—C3	1.352 (4)	C13—C14	1.415 (4)
C2—H2	0.9300	C14—C15	1.471 (4)
C3—C4	1.394 (5)	C15—N1	1.245 (3)
C3—H3	0.9300	C15—H15	0.9300
C4—C5	1.344 (5)	C16—C17	1.370 (4)
C4—H4	0.9300	C16—C21	1.393 (4)
C5—C6	1.430 (4)	C16—N1	1.414 (3)
C5—H5	0.9300	C17—C18	1.367 (4)
C6—C7	1.379 (4)	C17—H17	0.9300
C7—C8	1.399 (4)	C18—C19	1.361 (4)
C7—H7	0.9300	C18—H18	0.9300
C8—C9	1.415 (4)	C19—C20	1.364 (4)
C8—C13	1.430 (4)	C19—N2	1.466 (4)
C9—C10	1.341 (4)	C20—C21	1.385 (4)
C9—H9	0.9300	C20—H20	0.9300
C10—C11	1.412 (4)	C21—H21	0.9300
C10—H10	0.9300	N2—O1	1.212 (4)
C11—C12	1.342 (4)	N2—O2	1.216 (4)
			
C2—C1—C14	123.9 (2)	C11—C12—H12	119.2
C2—C1—C6	117.0 (2)	C13—C12—H12	119.2
C14—C1—C6	119.0 (2)	C14—C13—C12	124.0 (2)
C3—C2—C1	121.4 (3)	C14—C13—C8	119.4 (2)
C3—C2—H2	119.3	C12—C13—C8	116.6 (2)
C1—C2—H2	119.3	C13—C14—C1	120.3 (2)
C2—C3—C4	121.5 (3)	C13—C14—C15	123.2 (2)
C2—C3—H3	119.3	C1—C14—C15	116.5 (2)
C4—C3—H3	119.3	N1—C15—C14	126.8 (3)
C5—C4—C3	120.1 (3)	N1—C15—H15	116.6
C5—C4—H4	119.9	C14—C15—H15	116.6
C3—C4—H4	119.9	C17—C16—C21	119.2 (2)
C4—C5—C6	120.8 (3)	C17—C16—N1	117.1 (2)
C4—C5—H5	119.6	C21—C16—N1	123.6 (2)
C6—C5—H5	119.6	C18—C17—C16	120.9 (3)
C7—C6—C5	121.2 (3)	C18—C17—H17	119.6
C7—C6—C1	119.6 (2)	C16—C17—H17	119.6
C5—C6—C1	119.2 (3)	C19—C18—C17	118.9 (3)
C6—C7—C8	122.5 (2)	C19—C18—H18	120.5
C6—C7—H7	118.8	C17—C18—H18	120.5
C8—C7—H7	118.8	C18—C19—C20	122.6 (3)
C7—C8—C9	121.2 (2)	C18—C19—N2	118.3 (3)
C7—C8—C13	119.1 (2)	C20—C19—N2	119.1 (3)
C9—C8—C13	119.7 (2)	C19—C20—C21	118.2 (3)
C10—C9—C8	121.3 (3)	C19—C20—H20	120.9
C10—C9—H9	119.3	C21—C20—H20	120.9
C8—C9—H9	119.3	C20—C21—C16	120.1 (3)
C9—C10—C11	119.4 (3)	C20—C21—H21	119.9
C9—C10—H10	120.3	C16—C21—H21	119.9
C11—C10—H10	120.3	C15—N1—C16	120.0 (2)
C12—C11—C10	121.4 (3)	O1—N2—O2	123.0 (3)
C12—C11—H11	119.3	O1—N2—C19	118.2 (3)
C10—C11—H11	119.3	O2—N2—C19	118.7 (3)
C11—C12—C13	121.6 (3)		
			
C14—C1—C2—C3	−179.8 (2)	C8—C13—C14—C1	−1.7 (4)
C6—C1—C2—C3	−1.6 (4)	C12—C13—C14—C15	−4.1 (4)
C1—C2—C3—C4	0.1 (5)	C8—C13—C14—C15	177.7 (2)
C2—C3—C4—C5	1.1 (5)	C2—C1—C14—C13	177.9 (2)
C3—C4—C5—C6	−0.7 (5)	C6—C1—C14—C13	−0.2 (4)
C4—C5—C6—C7	177.4 (3)	C2—C1—C14—C15	−1.5 (4)
C4—C5—C6—C1	−0.8 (4)	C6—C1—C14—C15	−179.6 (2)
C2—C1—C6—C7	−176.3 (2)	C13—C14—C15—N1	−28.3 (4)
C14—C1—C6—C7	2.0 (4)	C1—C14—C15—N1	151.1 (3)
C2—C1—C6—C5	2.0 (4)	C21—C16—C17—C18	2.0 (5)
C14—C1—C6—C5	−179.8 (2)	N1—C16—C17—C18	179.4 (3)
C5—C6—C7—C8	179.9 (2)	C16—C17—C18—C19	−1.8 (5)
C1—C6—C7—C8	−1.8 (4)	C17—C18—C19—C20	1.2 (5)
C6—C7—C8—C9	−179.2 (2)	C17—C18—C19—N2	179.6 (3)
C6—C7—C8—C13	0.0 (4)	C18—C19—C20—C21	−0.7 (5)
C7—C8—C9—C10	176.7 (3)	N2—C19—C20—C21	−179.0 (2)
C13—C8—C9—C10	−2.4 (4)	C19—C20—C21—C16	0.8 (4)
C8—C9—C10—C11	0.4 (4)	C17—C16—C21—C20	−1.4 (4)
C9—C10—C11—C12	1.4 (5)	N1—C16—C21—C20	−178.6 (2)
C10—C11—C12—C13	−1.1 (5)	C14—C15—N1—C16	−179.1 (2)
C11—C12—C13—C14	−179.2 (2)	C17—C16—N1—C15	136.4 (3)
C11—C12—C13—C8	−1.0 (4)	C21—C16—N1—C15	−46.3 (4)
C7—C8—C13—C14	1.8 (4)	C18—C19—N2—O1	176.6 (3)
C9—C8—C13—C14	−179.0 (2)	C20—C19—N2—O1	−4.9 (5)
C7—C8—C13—C12	−176.5 (2)	C18—C19—N2—O2	−6.7 (5)
C9—C8—C13—C12	2.7 (4)	C20—C19—N2—O2	171.8 (3)
C12—C13—C14—C1	176.5 (2)		

### Hydrogen-bond geometry (Å, °)

**Table:**

Cg1 is the centroid of the C1–C6 benzene ring.

**Table:**

*D*—H···*A*	*D*—H	H···*A*	*D*···*A*	*D*—H···*A*
C12—H12···N1	0.93	2.37	2.980 (4)	123
C20—H20···Cg1^i^	0.93	2.86	3.717 (3)	154

Symmetry codes:  (i) −*x*+1, −*y*+2, −*z*.
